# Neighbourhood Walkability and Daily Steps in Adults with Type 2 Diabetes

**DOI:** 10.1371/journal.pone.0151544

**Published:** 2016-03-18

**Authors:** Samantha Hajna, Nancy A. Ross, Lawrence Joseph, Sam Harper, Kaberi Dasgupta

**Affiliations:** 1 Department of Epidemiology, Biostatistics and Occupational Health, McGill University, 1020 Pine Avenue West, Montréal, QC, Canada; 2 Department of Geography, McGill University, 805 Sherbrooke Street West, Montréal, QC, Canada; 3 Department of Medicine, Division of Clinical Epidemiology, McGill University Health Centre, 687 Pine Avenue West, Montréal, QC, Canada; University of Rome, ITALY

## Abstract

**Introduction:**

There is evidence that greater neighbourhood walkability (i.e., neighbourhoods with more amenities and well-connected streets) is associated with higher levels of total walking in Europe and in Asia, but it remains unclear if this association holds in the Canadian context and in chronic disease populations. We examined the relationships of different walkability measures to biosensor-assessed total walking (i.e., steps/day) in adults with type 2 diabetes living in Montreal (QC, Canada).

**Materials and Methods:**

Participants (60.5±10.4 years; 48.1% women) were recruited through McGill University-affiliated clinics (June 2006 to May 2008). Steps/day were assessed once per season for one year with pedometers. Neighbourhood walkability was evaluated through participant reports, in-field audits, Geographic Information Systems (GIS)-derived measures, and the Walk Score^®^. Relationships between walkability and daily steps were estimated using Bayesian longitudinal hierarchical linear regression models (n = 131).

**Results:**

Participants who reported living in the most compared to the least walkable neighbourhoods completed 1345 more steps/day (95% Credible Interval: 718, 1976; Quartiles 4 versus 1). Those living in the most compared to the least walkable neighbourhoods (based on GIS-derived walkability) completed 606 more steps per day (95% CrI: 8, 1203). No statistically significant associations with steps were observed for audit-assessed walkability or the Walk Score^®^.

**Conclusions:**

Adults with type 2 diabetes who perceived their neighbourhoods as more walkable accumulated more daily steps. This suggests that knowledge of local neighborhood features that enhance walking is a meaningful predictor of higher levels of walking and an important component of neighbourhood walkability.

## Introduction

Higher neighbourhood walkability (i.e., the ‘walking friendliness’ of a neighbourhood) has been linked to higher levels of biosensor-assessed total walking in Europe and in Asia [1], but there is evidence that this association may be null in the Canadian context [2]. Since adults living with chronic diseases face a unique set of challenges to engaging in physical activity, they may be particularly sensitive to features of their neighbourhood environments [3, 4]. Adults with type 2 diabetes are a group of individuals who are particularly inactive and unmotivated to engage in physical activity [5–13]. Several studies have demonstrated positive associations between neighbourhood walkability and physical activity in this population [4, 5, 14], but to our knowledge, no studies have been conducted in North America using biosensor-assessed measures of total walking. From a socio-ecological perspective, it is important to understand the influence of the environment on walking levels in this high-risk, sedentary group of individuals.

Neighbourhood walkability can be assessed using participant-reported (i.e., perceived) measures of walkability, in-field or virtual street-level audits (e.g., Google Street View), and publicly available measures (e.g., Walk Score®). Geographic Information Systems (GIS)—digital methods for processing large amounts of spatial data [15]–represents one of the most common ways that neighbourhood walkability is assessed for research purposes. Using GIS, walkability is often operationalized based on a neighbourhood’s street connectivity, residential and/or population density, and land use mix. Street connectivity is commonly defined as the number of intersections within a given area. More intersections facilitate movement between origins and destinations [16, 17]. Residential and/or population density are defined as the number of people and/or residences within a given area [18]. Areas with greater residential/population densities are generally more conducive to non-motorized transport as a result of there being more people to visit and a greater demand for accessible community services, such as shops and parks [16]. Land use mix is a measure of the evenness of the distribution of the land uses within a neighbourhood [17, 19]. The more types of land uses that are contained within a neighbourhood, the more convenient it is to walk to services supplied by these areas [20, 21].

We previously demonstrated a strong correlation between GIS-derived walkability (based on street connectivity, residential density and land use mix) and an overall index of neighbourhood walkability as captured by an in-field audit [22]. Neither of these measures, however, correlated well with participant-reported walkability [22]. In the present follow-up analysis, we examined the relationships of these three walkability measures to daily steps in a sample of adults living with type 2 diabetes (QC, Canada) on whom we had repeated-measures of pedometer-assessed walking over a one-year period. To our knowledge, no previous study has concurrently examined the relationships of these different walkability measures with daily steps. The objective of this study was to improve our understanding of how neighbourhood environments might influence the physical behaviours of adults living with type 2 diabetes.

We were specifically interested in the association between neighbourhood walkability and daily steps, as opposed to other forms of physical activity (e.g., moderate-to-vigorous intensity physical activity (MVPA)) for two reasons. First, pedometer-assessed daily steps are an accurate measure of total habitual walking in adults [23–25] that have been linked to important health benefits in adults with type 2 diabetes. For example, in a sample of over 9,000 adults with impaired glucose tolerance), pedometer-assessed steps at baseline ((Hazard Ratio) HR for a 2,000 steps/day increment = 0.90, 95% CI 0.84, 0.96) and change in steps over an average follow-up of six years (HR per 2000 steps/day increase = 0.92, 95% CI 0.86, 0.99) led to reductions in cardiovascular disease events, (i.e., cardiovascular mortality, stroke, or myocardial infarction) [26]. Second, since walking is the most common and preferred form of physical activity among adults [27–30], understanding its link to neighbourhood walkability, as opposed to other forms of physical activity, may have population-wide benefits.

## Materials and Methods

### Study population

Adults (n = 201) with physician-diagnosed type 2 diabetes were recruited through McGill-affiliated outpatient clinics (Montreal, QC) and local diabetes associations between June 2006 and May 2008. They attended four in-clinic assessments, one per season, over the course of one year [31]. As previously described [31, 32], to allow for accurate measurements of steps using pedometers, participants were required to have a normal gait and a body mass index (BMI) of less than 40 kg/m^2^. Those who were pregnant or planning a pregnancy were ineligible, as were those with chronic conditions that could compromise glycemic control. Procedures were approved by McGill University’s Faculty of Medicine Institutional Review Board and all participating institutions. Participants provided written informed consent. Written informed consent was recorded using a consent form and procedure that was approved by McGill University's Faculty of Medicine Institutional Review Board.

### Measures

#### Daily steps

Daily steps were assessed once per season for 14 consecutive days using Yamax SW-701 pedometers with viewing windows concealed [33]. A pedometer with the same step counting mechanism (i.e., the SW-701 model) has been shown to count steps to within 3% of actual steps taken [25, 31, 33]. Participants were provided with three pedometers: A and B were each worn for a seven-day period; C remained in the postage-paid envelope and accounted for extra steps accumulated during the mailing process. Mean daily steps were calculated by dividing the total number of steps accumulated on Pedometers A and B (corrected for the steps accumulated on Pedometer C) by the total number of days the pedometers were worn. In the event that some participants would not be able to wear their pedometers for the full 14-day period, we provided all participants with a form on which they could indicate their wear days.

#### Participant-reported walkability

Three surveys of social and physical environments have been shown to have good test-retest reliability [31, 34]. In our study questionnaire, we included the items from these surveys that were relevant to our outcome of interest (i.e., walking). The items that we queried included presence/condition of sidewalks, street lighting, traffic, proximity to stores and transit stops, presence of interesting sights, activity level of neighbours, and safety while walking. Based upon the participants’ responses to these items, we calculated participant-reported walkability as the sum of the regression-based scores calculated for the factors that we identified via a principal component analysis [35]. A higher score indicated greater walkability.

#### GIS-derived walkability

Residential neighbourhoods were defined as 500-meter polygonal street network buffers around the centroid of each participant’s home postal code address. Street connectivity, residential density and land use mix were calculated within these neighbourhoods using GIS (ArcGIS 10.1; ESRI; Redlands, CA). Street connectivity was measured as the number of ≥4-way intersections per square kilometer. Residential density was equivalent to the number of residences per square kilometer of residential land area. Land use mix represented the degree of heterogeneity in residential, commercial, institutional and recreational land uses and was equal to (-1) x [((proportion of residential land) ln (proportion of residential land)) + ((proportion of commercial land) ln (proportion of commercial land)) + ((proportion of institutional land) ln (proportion of institutional land)) + ((proportion of recreational land) ln (proportion of recreational land))] / ln 4. The land use mix score ranged from 0 to 1. A higher score indicated a greater mixing of land uses within a neighbourhood. Land use mix and street connectivity were calculated based on data obtained from the 2008 DMTI Quebec land use and Montreal road segment files [36, 37]. Residential density was calculated using data obtained from 2006 Canadian Census files [38]. In line with previous methods [17, 19, 35, 39], GIS-derived walkability was calculated by summing the z-scores of street connectivity, residential density and land use mix. A higher score indicated greater overall neighbourhood walkability based on these three measures. We have previously validated this measure in this study population against neighbourhood walkability assessed via the in-field audit that we describe below (R = 0.7, 95% CI 0.6, 0.8) [22].

#### Audit-assessed walkability

Five randomly-selected street segments within 500-meters of each participant’s home postal code were audited in 2009 using a 21-item modified version of the Pedestrian Environment Data Scan (PEDS) [40]. PEDS has been shown to be a reliable tool for the assessment of pedestrian environments [40]. Audit-assessed walkability was quantified as the sum of the regression-based scores calculated for the factors identified via a principal component analysis [22]. A higher score indicated greater walkability.

#### Walk Score^®^

The Walk Score^®^ is a validated measure that captures the walkability of a geographic location based on its proximity to 13 walkable destinations (e.g., stores) using a publicly available interface (www.walkscore.com) [41, 42]. The score ranges from 0 (car-dependent) to 100 (walker’s paradise) and is calculated based on an algorithm that assigns equal weights to each of the walkable destinations [41, 42].

#### Covariates

Age, sex, insulin use, annual household income (≥$50,000), married/common-law, university education, ethnicity, immigrant status, smoking status, dog ownership, and diabetes duration were reported by participants at baseline. BMI was computed from direct weight and height measurements taken at baseline. Depressed mood was assessed at each visit using the Center for Epidemiologic Studies-Depression Scale (CES-D Score ≥16) [43, 44]. Residential self-selection (11-items from the Neighbourhood Quality of Life Study questionnaire [45]), vehicle access, years living at address, and past participation in regular physical activity were ascertained as part of a follow-up survey mailed to participants in the winter of 2012/2013. Season was based on visit date and corresponded to solstice calendar definitions of fall, winter, spring and summer (e.g., fall: September 22/23 to December 20/21). Because steps were similar in the spring and summer and in the fall and winter [32], seasons were dichotomized into spring/summer and fall/winter categories.

### Statistical analysis

Descriptive statistics were produced for all variables of interest overall and by quartile of GIS-derived neighbourhood walkability. Spearman correlation coefficients and scatter plots were produced for the associations between steps, participant-reported walkability, GIS-derived walkability, audit-assessed walkability and the Walk Score^®^. Given repeated (seasonal) measures of steps, Bayesian hierarchical linear regression models with diffuse priors were used to estimate the associations between the measures of walkability (across quartiles) and steps over time (WinBUGS 1.4.3). Associations between season and steps were assessed at concurrent time points. Data on residential self-selection, vehicle access, years living at address, and past participation in physical activity were available on only a subgroup of participants who completed the follow-up survey (n = 78). Because of the influence on sample size and the fact that adjustment for these variables did not appear to lead to important changes to the main estimates of interest (i.e., walkability and daily steps), we did not include them in the final models. Final models were based on complete case data (n = 131). Variables were selected into the models based on theoretical importance and/or if they were identified (based on univariate and correlation analyses) as potential confounders or predictors of daily steps. The interpretation of findings was based on 95% credible intervals (CrI), the Bayesian analog of frequentist confidence intervals (CI). All analyses were conducted in 2014.

## Results

### Descriptive statistics

Sixty-nine percent (69.2%) of participants attended all four visits with over 84.1% attending three visits. Of the 688 visits attended, 182 occurred in spring (26.5%), 165 in summer (24.0%), 185 in fall (26.9%), and 156 in winter (22.7%). 174 participants (86.6%) were evaluated at least once during both the spring/summer and the fall/winter periods. Of the 201 participants enrolled, 108 participants returned the mailed questionnaire. Of these, 78 (38.8%) provided complete data on all four additional covariates of interest, including residential self-selection, vehicle access, years living at address, and past physical activity.

Participants (mean = 60.5 years, standard deviation (SD) = 10.4) averaged 5388 steps/day (SD = 2488). The most walkable neighbourhoods (i.e., Quartile 4) had the lowest proportion of married couples, people having annual household incomes of more than $50,000 per year, and people with regular access to a vehicle (Table 1). A negative graded association was observed between neighbourhood walkability and regular vehicle access with those living in less walkable neighbourhoods, having greater regular access to a vehicle (Q1: 92.9%; Q2: 88.9%; Q3: 86.7%; Q4: 55.0%). The most walkable neighbourhoods contained the highest proportion of immigrants (Table 1). There was no discernable pattern in daily steps across quartiles of neighbourhood walkability (Table 1). On average, neighbourhoods were “somewhat walkable” based on the Walk Score^®^ definition of walkability (Walk Score^®^ = 69, SD = 19). There was good variability in neighbourhood walkability as assessed by the Walk Score^®^ with 17.5% of the study population living in “car dependent” neighbourhoods (i.e., Walk Scores^®^<49) and 12.2% of the study population living in “very walkable/Walker’s paradise” neighbourhoods (Table 2). Some differences were observed between completers and non-completers of the follow-up survey (e.g., more women and university educated adults completed the follow-up survey, S1 Table) and between participants included and excluded from the final models (e.g., more women and adults earning ≥$50,000 per year were included in the final models, S2 Table).

**Table 1:** Characteristics of the study population at baseline by quartile of neighbourhood walkability (n = 131).^a,b^

		Neighbourhood walkability^a^			
	Overall^b^	Quartile 1	Quartile 2	Quartile 3	Quartile 4
	mean (SD)	mean (SD)	mean (SD)	mean (SD)	mean (SD)
Age, *years*	60.5 (10.4)	60.8 (9.5)	63.0 (9.8)	58.9 (11.9)	59.2 (10.0)
Body mass index, *kg/m*^*2*^	30.3 (5.8)	30.4 (6.1)	29.0 (5.9)	31.6 (5.3)	30.1 (5.8)
Daily steps	5388 (2488)	5121 (2593)	5828 (2462)	4816 (2468)	5764 (2397)
Walk Score^®^	69 (19)	48 (15)	64 (15)	79 (10)	84 (12)
	**%**	**%**	**%**	**%**	**%**
Women	48.1	37.5	21.2	68.8	35.3
Married/common-law	69.5	87.5	78.8	56.3	55.9
University education	38.2	40.6	42.4	13.3	38.2
Annual household income, *≥$50*,*000*	45.3	60.7	57.1	34.5	31.3
Ethnicity, *White*	71.0	68.8	69.7	75.0	70.6
Immigrant	45.0	43.8	42.4	37.5	55.9
Depressed mood	28.2	28.1	12.2	37.5	35.3
Dog ownership	14.5	21.9	6.1	18.8	11.8
Insulin use	34.4	40.6	36.4	40.6	20.6
Regular vehicle access	79.1	92.9	88.9	86.7	55.0
Past regular exercise	80.6	78.6	83.3	93.3	70.0

^a^ Quartile cut-offs for the GIS-derived walkability index: Quartile 1: < -2.17 (n = 32); Quartile 2: ≥-2.17<0.13 (n = 33); Quartile 3: ≥0.13<1.67 (n = 32); Quartile 4: ≥1.67 (n = 34); Q1: annual household income (n = 28), regular vehicle access and past regular exercise (n = 14); Q2: daily steps (n = 32), annual household income (n = 28), regular vehicle access and past regular exercise (n = 18); Q3: Annual household income (n = 29), regular vehicle access and past regular exercise (n = 15); Q4: Annual household income (n = 32), regular vehicle access and past regular exercise (n = 20).

^b^ Daily steps (n = 130); annual household income (n = 117); regular vehicle access and past regular exercise (n = 67).

**Table 2:** The distribution of neighbourhood walkability (based on the Walk Score^®^)^a^ in the study population. (n = 131).

Walk Score^®^	Walk Score Category^®^	% (n)
90–100	Walker’s Paradise *(Daily errands do not require a car)*	12.2% (16)
70–89	Very Walkable *(Most errands can be accomplished on foot)*	45.0% (59)
50–69	Somewhat walkable *(Some errands can be accomplished on foot)*	25.2% (33)
25–49	Car-dependent *(Most errands require a car)*	16.0% (21)
0–24	Car-dependent *(Almost all errands require a car)*	1.5% (2)

^a^ Categories and descriptions are taken directly from www.walkscore.com

### Correlation analyses

The Walk Score^®^ correlated moderately with audit-assessed walkability (R = 0.5, 95% CI 0.3, 0.6; n = 201) and GIS-derived walkability (R = 0.8, 95% CI 0.7, 0.8; n = 200) and minimally with participant-reported walkability (R = 0.1, 95% CI -0.01, 0.3; n = 200). The correlations among the other measures have been reported previously (audit/GIS: R = 0.7, 95% CI 0.6, 0.8; participant-reported/audit: R = 0.2, 95% CI 0.1, 0.3; participant-reported/GIS: R = 0.2, 95% CI 0.04, 0.3) [22]. Scatter plots between the four walkability measures and steps are provided in S1 and S2 Figs. A small correlation was observed between steps and participant-reported walkability (R = 0.2, 95% CI 0.1, 0.3; n = 194, S2A Fig). There was very little relation between steps and the other walkability measures (S2B–S2D Fig).

### Multivariate models

#### Participant-reported walkability

Adults who reported living in the most compared to the least walkable neighbourhoods completed 1345 more steps/day (95% CrI: 718, 1976). There were no important differences for the first through third quartiles (Table 3).

**Table 3:** Mean differences in daily steps across quartiles of each of the measures of neighbourhood walkability (n = 131).

	Increment in Daily Steps (95% credible interval)^a^^,^^b^		
	**Quartile 2**	**Quartile 3**	**Quartile 4**
**Participant-reported walkability**	*mean = -0*.*4*, *SD = 0*.*4*	*mean = 0*.*7*, *SD = 0*.*2*	*mean = 1*.*9*, *SD = 0*.*7*
*Model 1*	34 (-1050, 1103)	-393 (-1545, 768)	1344 (88, 2572)
*Model 2*	122 (-440, 688)	-189 (-774, 408)	1364 (733, 1990)
*Model 3*	103 (-457, 677)	-197 (-774, 395)	1345 (718, 1976)
	**Quartile 2**	**Quartile 3**	**Quartile 4**
**GIS-derived walkability**	*mean = -0*.*9*, *SD = 0*.*7*	*mean = 1*.*0*, *SD = 0*.*5*	*mean = 3*.*0*, *SD = 1*.*2*
*Model 1*	970 (-188, 2133)	143 (-990, 1276)	794 (-354, 1976)
*Model 2*	1011 (412, 1604)	57 (-550, 653)	724 (130, 1314)
*Model 3*	783 (168, 1406)	-30 (-616, 557)	606 (8, 1203)
	**Quartile 2**	**Quartile 3**	**Quartile 4**
**Audit-assessed walkability**	*mean = -0*.*6*, *SD = 0*.*3*	*mean = 0*.*5*, *SD = 0*.*4*	*mean = 2*.*5*, *SD = 1*.*3*
*Model 1*	-214 (-1364, 941)	-279 (-1441, 899)	-410 (-1608, 811)
*Model 2*	-325 (-916, 264)	119 (-481, 713)	-87 (-699, 507)
*Model 3*	-157 (-753, 431)	39 (-556, 633)	-240 (-834, 359)
	**Quartile 2**	**Quartile 3**	**Quartile 4**
**Walk Score**^**®**^	*mean = 61*, *SD = 5*	*mean = 76*, *SD = 4*	*mean = 89*, *SD = 6*
*Model 1*	-723 (-1954, 505)	-642 (-1826, 565)	-127 (-1257, 1044)
*Model 2*	255 (-393, 895)	-241 (-854, 381)	7 (-577, 600)
*Model 3*	114 (-524, 769)	-232 (-834, 360)	-204 (-782, 381)

^a^ Quartile 1 served as the reference. (Quartile 1 means (standard deviations, SD): participant-reported walkability = -2.0 (SD 0.9); GIS-derived walkability = -3.0 (SD 0.6); audit-assessed walkability = -2.1 (SD 0.6); Walk Score^**®**^ **=** 42 (SD 10))

^b^ **Model 1:** Unadjusted; **Model 2:** Adjusted for age, sex, BMI, depressed mood, dog ownership, insulin use, immigrant status, and season; **Model 3 (participant-reported walkability):** Adjusted for age, sex, BMI, depressed mood, dog ownership, insulin use, immigrant status, season, and GIS-derived walkability; **Model 3 (GIS-derived walkability, audit-assessed walkability, and Walk Score**^**®**^**):** Adjusted for age, sex, BMI, depressed mood, dog ownership, insulin use, immigrant status, season, and participant-reported walkability.

#### GIS-derived walkability

Those living in the most compared to the least walkable neighbourhoods (Q4 versus Q1) completed 606 more steps per day (95% CrI: 8, 1203). The difference in steps between the second and first quartiles was similar in magnitude (783 more steps/day for the second quartile, 95% CrI: 168, 1406). Quartile 3 demonstrated no important differences with Quartile 1.

#### Audit-assessed walkability

No statistically significant association was observed for audit-assessed walkability and daily steps. The point estimates suggested a negative association (e.g., Model 3 Quartile 4 versus 1: -240 steps/day, 95% CI -834, 359), but the confidence intervals included zero (Table 3).

#### Walk Score^®^

Similar to audit-assessed walkability, no statistically significant association was observed for the Walk Score^®^ (e.g., Model 3: Quartile 4 versus 1: -204 steps/day, 95% CI -782, 381; Table 3).

#### Other correlates of daily steps

While several potentially important predictors of daily steps emerged in univariate models (S3 Table), the factors that remained important in the fully adjusted model (i.e., adjusted for age, sex, BMI, depressed mood, dog ownership, insulin use, immigrant status, season, GIS-derived neighbourhood walkability, and participant-reported neighbourhood walkability; S4 Table) included age, BMI, absence of depressed mood, dog ownership, and summer/spring season. Every one-year decrement in age was associated with 106 more steps/day (95% CrI: 85, 127), every one-unit decrement in BMI was associated with 119 more steps/day (95% CrI: 82, 155), and absence of depressed mood was associated with 553 more steps/day (95% CrI: 90, 1023). Dog owners completed 646 more steps/day (95% CrI: 28, 1250). Participants completed 692 steps/day in the summer/spring compared to the fall/winter (95% CrI: 283, 1106).

## Discussion

We examined the associations between multiple measures of walkability with daily steps in a sample of adults with type 2 diabetes. Our findings demonstrate that those individuals who gave a more favorable assessment of their neighbourhood’s walkability took 1345 more steps per day than those individuals who had a less favorable assessment (Quartile 4 versus 1; 95% CI 718, 1976). This is equivalent to approximately 13.5% of the recommended steps per day. Although we found a positive association between neighbourhood walkability and daily steps for the second and fourth quartiles of GIS-derived neighbourhood walkability, more studies are needed to determine if these associations are clinically important. No important associations were observed for audit-assessed walkability or the Walk Score^®^. We identified several other important predictors of higher levels of walking among adults with type 2 diabetes. These included a demonstrable effect of absence of depressed mood, dog ownership and spring/summer (compared to fall/winter) season.

Our findings on the relationship between participant-reported walkability and daily steps are consistent with the recently published results from the 11-country International Physical Activity and the Environment Network (IPEN) Adult Study. In this study individuals who reported easier access to destinations and services were 17% more likely to achieve ≥420 minutes/week of MVPA, those who reported better neighbourhood aesthetics were 13% more likely to achieve ≥420 minutes/week of MVPA, and those who reported greater safety from crime were 14% more likely to achieve ≥420 minutes/week of MVPA [46].

We demonstrated that there is a beneficial association between GIS-derived neighbourhood walkability and daily steps in adults with type 2 diabetes. It remains unclear, however, if these benefits are clinically important. We did not find any important associations with daily steps for audit-based walkability or the Walk Score^®^. A possible explanation for a positive association for GIS-derived neighbourhood walkability but not for these two measures is that audit-based walkability and the Walk Score^®^ capture different characteristics of home neighbourhoods. The audit-based walkability index captured finer-scale features of a neighbourhood environment (e.g., crossing aids and sidewalk conditions), and the Walk Score^®^ captured the proximity of homes to 13 walkable destinations. This is in contrast to the GIS-derived measure of walkability, which captured three large-scale characteristics of urban designs (i.e., street connectivity, residential density and land use mix). It is possible that, in this population, larger-scale rather than finer-scale features of neighbourhoods may play slightly more of an important role in the total amount of walking that adults with type 2 diabetes achieve.

Our finding of a clear positive association for perceived neighbourhood walkability and a less clear association for more objective measures of neighbourhood walkability (i.e., GIS-derived, audit-based, and the Walk Score^®^) is in line with the pervious work. It has been estimated that there is a 30% mismatch between perceived and objectively assessed walkability [47, 48] and that the correlation between these measures is low [22, 47]. This suggests that these measures are capturing different aspects of walkability and thus, it is not unexpected that they would have different relationships with the same outcome of interest. Indeed, there is evidence of this elsewhere in the literature. For example, in a recent study of 5124 adults who were free of type 2 diabetes at baseline and who participated in the Multi-Ethnic Study of Atherosclerosis, participant-reported neighbourhood walkability (based on resources that support physical activity) was more strongly associated with lower risk of incident type 2 diabetes over 8.9 years of follow-up than GIS-derived neighbourhood walkability (i.e., HR = 0.79, 95% CI 0.71, 0.88 *versus* HR = 0.96, 95% CI 0.92, 0.99) [49].

In addition to better perceived (i.e., participant-reported) neighbourhood walkability, absence of depressed mood, dog ownership, and spring/summer (versus fall/winter) season were identified as important predictors of higher daily steps in adults with type 2 diabetes. Approximately one fourth of women and one sixth of men with diabetes have depressive symptoms [50, 51]. In a study of 2,646 primary care patients with type 2 diabetes, depressed patients were nearly two times more likely to be inactive than non-depressed patients (Odds Ratio (OR) = 1.74, 95% CI: 1.32, 2.31) [52]. There is also evidence that higher levels of physical activity may lead to lower risk of incident depression. In a study of 1,947 older community-dwelling adults, higher physical activity was associated with a 17% decreased likelihood of developing depression over five years (OR = 0.83, 95% CI: 0.73, 0.96) [53]. Our study is the first to quantify the association between depressed mood and biosensor-assessed daily steps in patients type 2 diabetes. We found that absence of depressed mood was associated with taking 553 more steps/day (95% CrI 90, 1023). Although we cannot draw conclusions regarding causality or directionality of the relationship, treating depressive symptoms might lead to increases in walking and/or facilitating increases in walking (e.g., by prescribing daily steps [54]) might alleviate symptoms of depression in adults with type 2 diabetes. In line with previous findings [55], we also determined that dog owners achieved 646 steps/day (95% CrI: 28, 1250) more than non-dog owners. Based on this, and evidence that encouraging dog walking among dog owners may increase their daily steps [56, 57], promoting dog walking may be an important point to leverage especially in populations where dog ownership may be high. The seasonal differences in daily steps that we identified were similar to those described in other studies [58–60]. In a previous analysis of this cohort, we demonstrated a -758 mean fall/winter to spring/summer difference in daily steps (95% CI -1037, -479) [32]. In this study, we confirmed that the association held independently of several covariates, including walkability. Given fall/winter declines in walking, public health and clinical strategies need to encourage and support maintenance of physical activity levels in fall and winter months.

We demonstrated that a high percentage of participants who completed the mailed questionnaire had regular access to a vehicle a car (79.1%) and that there is a negative graded association between neighbourhood walkability and regular vehicle access. We also demonstrated that respondents who had regular vehicle access accumulated 1426 fewer steps/day (95% CI -2752, -118) than respondents who did not have regular vehicle access (based on univariate linear regression analyses, S3 Table). Given a clear association between regular vehicle access and lower daily steps, discouraging reliance on cars may be a way to facilitate increases in physical activity. Even though including vehicle access in our models did not appear to alter our conclusions, it should be noted that we did not have enough data on vehicle access in order to fully investigate the role of this variable. To understand the role of vehicle access on the walkability-physical activity relationship in adults with type 2 diabetes, other studies will need to be conducted. Of particular interest are the mediating and moderating roles of vehicle access. There have been some studies conducted on the moderating and/or mediating roles of vehicle access in general adult and older adult populations [61, 62]. In a study of 2178 Swedish adults, vehicle access mediated 25% of the association between residential density and accelerometer-assessed MVPA and 34% of the association between land use mix and accelerometer-assessed MVPA [62]. Although vehicle access does not appear be an important moderator of the neighbourhood walkability-physical activity relationship in some studies [61, 62], it does in others [63, 64]. Differences are likely due to study populations and/or differences in exposure and outcome measurement [62].

Strengths of this study included objective assessments of exposures and outcome, assessment of residential self-selection multiple measures of walkability, and repeated measurements of daily steps over time. Repeated outcome measures increase the power to detect effects [65, 66]. An added strength was that our study is the first to examine the link between GIS-derived walkability and daily steps in North America adults with type 2 diabetes. Daily steps are of particular interest as they are more easily understood by patients and practitioners than activity counts or time spent in MVPA. It is important to note, however, that had we used another outcome (e.g., MVPA), it is possible that important associations may have emerged. We acknowledge some potential limitations. First, we cannot be definitive about the directionality or causality of the relationships. Because follow-up did not commence with the ‘onset’ of moving to a walkable neighbourhood, we cannot conclude that walkability led to higher steps. It remains possible that more active people perceive their neighbourhoods as more walkable and/or move to neighbourhoods that are. Second, we cannot make definitive conclusions regarding the neighbourhood walkability-walking relationship independent of vehicle access and residential self-selection. We collected these data in follow-up to an already completed study and thus were only able to obtain this information on a subsample of our study population. Our analyses regarding the role of these variables were exploratory. Third, our overall sample size limited the accuracy of the estimated effects. More definitive conclusions could be drawn had more data been available. Fourth, walkability cannot influence steps if one is not exposed to the environment. Although studies on location-based physical activity are emerging [67–69], more studies using Geographical Positioning Systems monitoring are needed to make a definitive connection between environmental exposure and behaviour [61]. Lastly, because differences in socio-demographic characteristics were observed between participants included and excluded from the final analyses (e.g., annual household income, ethnicity), the possibility of selection bias cannot be excluded.

## Conclusions

Despite these potential limitations, there are some important conclusions that can be drawn from these analyses. Participant-reported walkability appears to be an important predictor of daily steps in adults with type 2 diabetes. There is a positive association between neighbourhood walkability and daily steps for the second and fourth quartiles of GIS-derived neighbourhood walkability, but more studies are needed to determine if these associations are clinically important. No important associations were observed for audit-assessed walkability or the Walk Score^®^. Residents’ knowledge of neighbourhood features is a meaningful component of the concept of walkability and publicizing features that enhance walkability may lead to improvements in perceptions and ultimately higher daily steps. Season was confirmed to be an important predictor of daily steps as were several individual-level factors, including absence of depressed mood and dog ownership. Developing strategies that address individual-level and environmental factors in combination may prove useful for facilitating increases in total walking.

## Supporting Information

S1 Fig Scatter plots comparing the four walkability measures of interest. (DOCX)

S2 Fig Scatter plots of daily steps by each of the four walkability measures of interest. (DOCX)

S1 Table Characteristics of participants who did and did not complete the follow-up survey. (DOCX)

S2 Table Characteristics of the participants that were included and excluded from the final models. (DOCX)

S3 Table Univariate longitudinal hierarchical linear regression estimates between the covariates of interest and daily steps (n = 131).^a,b^ (DOCX)

S4 Table Fully adjusted hierarchical longitudinal linear regression model for the association between participant-reported neighborhood walkability and daily steps (n = 131). (DOCX)
