# Supplementary material for: Neighbourhood Walkability and Daily Steps in Adults with Type 2 Diabetes
Source: PLoS One. 2016 Mar 18;11(3):e0151544. doi: 10.1371/journal.pone.0151544 (PMC4798718; doi:10.1371/journal.pone.0151544)
Supplement: S1 Fig — (DOCX) [file pone.0151544.s001.docx]

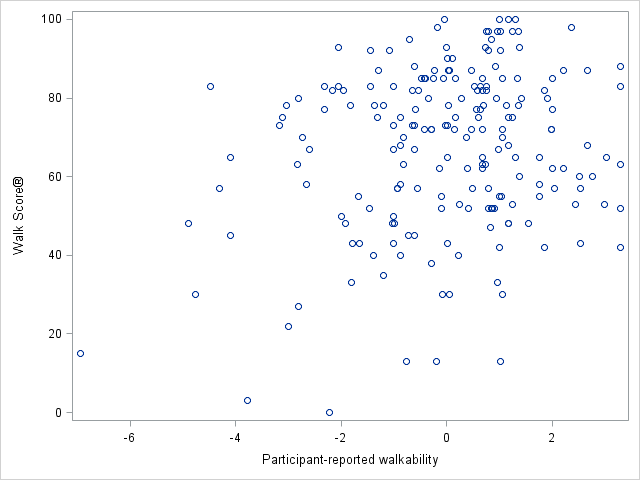

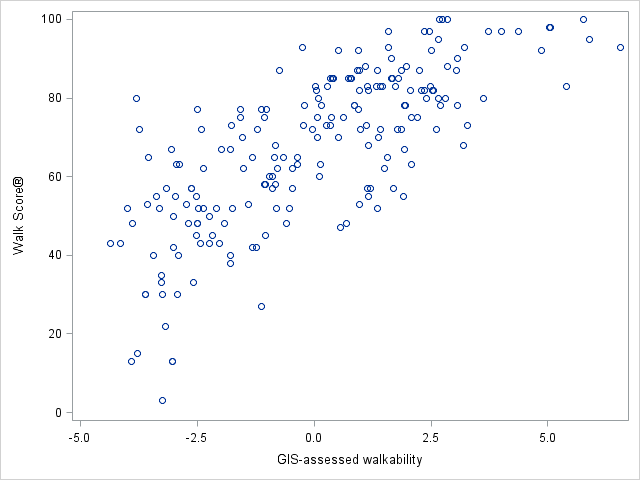


a) GIS-derived walkability *vs.* Walk Score® b) Participant-reported walkability *vs.* Walk Score®


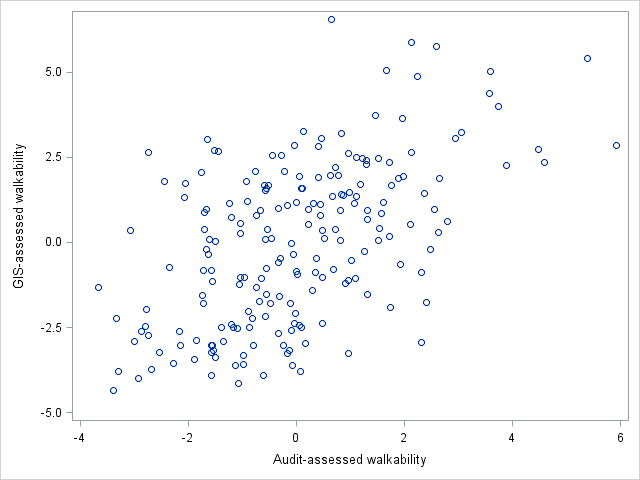

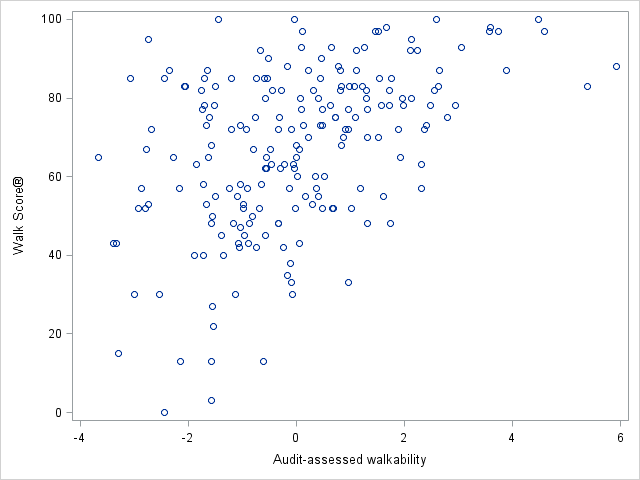


c) Audit-assessed walkability *vs.* Walk Score® d) Audit-assessed walkability *vs.*GIS-derived walkability


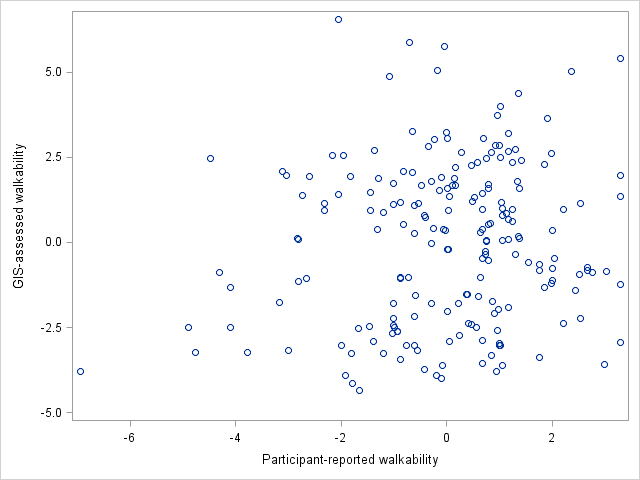

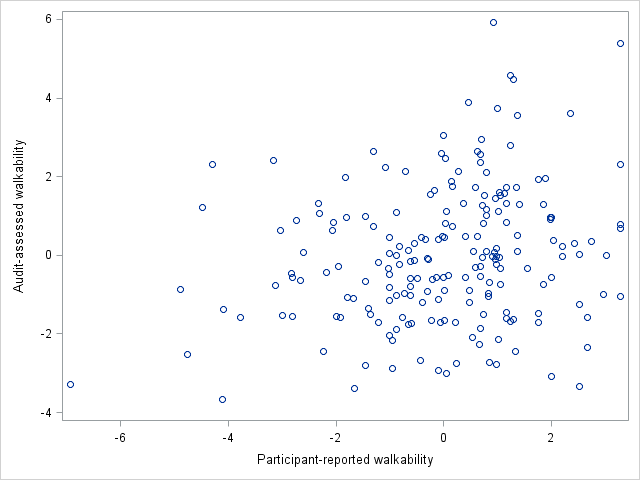


e) Participant-reported walkability *vs.*audit-assessed f) Participant-reported walkability *vs.*GIS-derived walkability

walkability

**S1 Fig. Scatter plots comparing the four walkability measures of interest.**
