# Supplementary material for: Neighbourhood Walkability and Daily Steps in Adults with Type 2 Diabetes
Source: PLoS One. 2016 Mar 18;11(3):e0151544. doi: 10.1371/journal.pone.0151544 (PMC4798718; doi:10.1371/journal.pone.0151544)
Supplement: S2 Fig — (DOCX) [file pone.0151544.s002.docx]

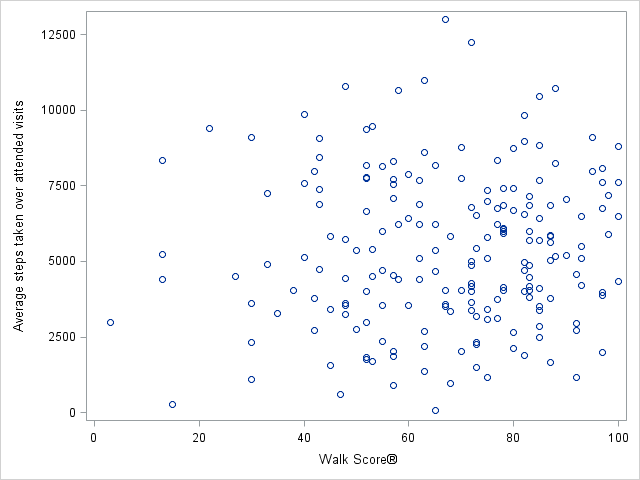

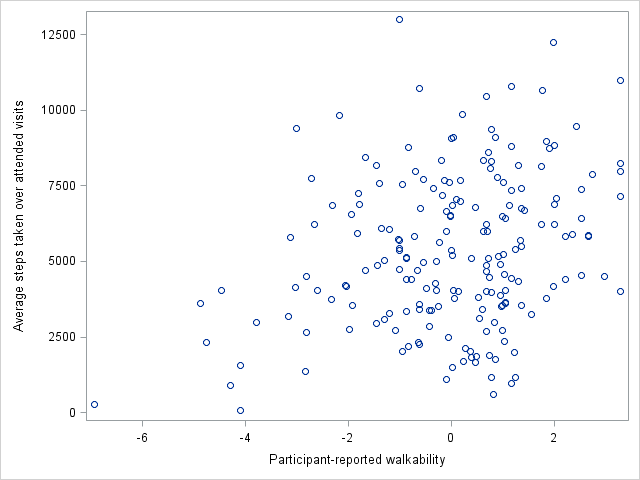


a) Participant-reported walkability *vs.* daily steps b) Walk Score® *vs.*daily steps


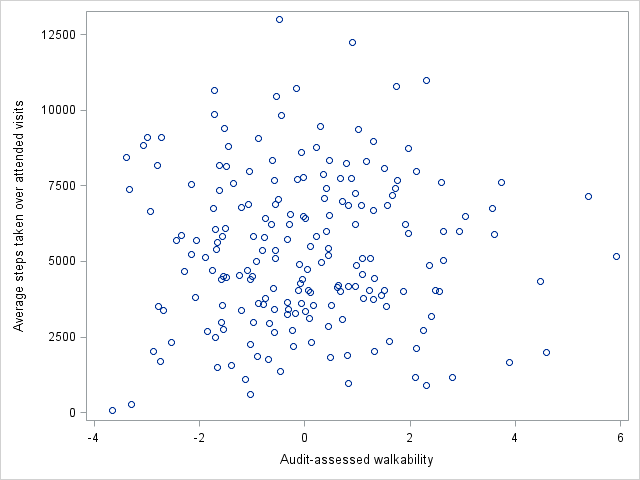

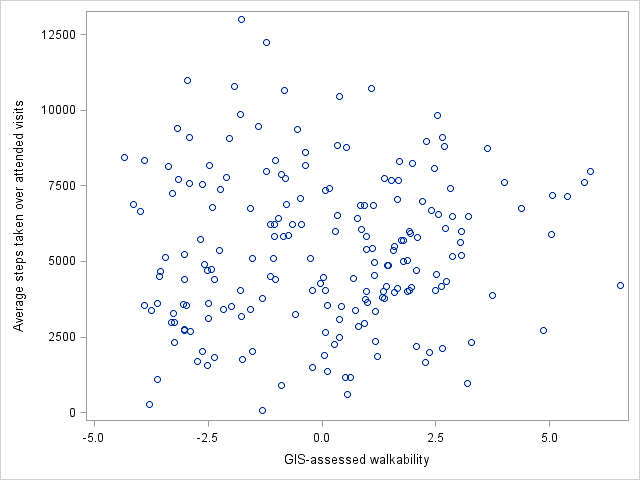


c) GIS-derived walkability *vs.* daily steps d) Audit-assessed walkability *vs.*daily steps

**S2 Fig. Scatter plots of daily steps by each of the four walkability measures of interest.**
