# Supplementary material for: Neighbourhood Walkability and Daily Steps in Adults with Type 2 Diabetes
Source: PLoS One. 2016 Mar 18;11(3):e0151544. doi: 10.1371/journal.pone.0151544 (PMC4798718; doi:10.1371/journal.pone.0151544)
Supplement: S1 Table — (DOCX) [file pone.0151544.s003.docx]

**S1 Table. Characteristics of participants who did and did not complete the follow-up survey.**

|  | **Complete Follow-up Survey^a^ (n=78)** | **Incomplete Follow-up Survey ^b^ (n=123)** |
| --- | --- | --- |
|  | **mean (SD)** | **mean (SD)** |
| Age, *years* | 59.6 (9.9) | 60.1 (10.9) |
| Steps/day | 5484 (2456) | 5287 (2785) |
| Body mass index, *kg/m^2^* | 30.2 (5.6) | 30.6 (5.6) |
| Diabetes duration, *years* | 9.0 (9.3) | 9.7 (7.1) |
| Participant-reported walkability | 0.3 (1.5) | -0.2 (1.9) |
| GIS-derived walkability | -0.1 (2.2) | 0.04 (2.5) |
| Audit-assessed walkability | 0.1 (1.7) | -0.1 (1.7) |
| Walk Score^®^ | 65.1 (21.3) | 68.4 (20.1) |
|  |  |  |
|  | **n (%)** | **n (%)** |
| Women | 42 (53.9) | 52 (42.3) |
| Married/common-law | 52 (70.3) | 72 (67.9) |
| University education | 39 (50.0) | 39 (31.7) |
| Annual household income, *≥ $50,000* | 41 (56.9) | 36 (34.0) |
| Ethnicity, *white* | 56 (71.8) | 83 (67.5) |
| Immigrant | 30 (38.5) | 63 (51.2) |
| Current smoking | 9 (12.3) | 8 (7.6) |
| Insulin use | 24 (30.8) | 42 (34.2) |
| Depressed mood | 18 (23.4) | 37 (30.1) |
| Dog ownership | 10 (12.8) | 21 (17.1) |

^a^ Married/common-law (n=74); annual household income (≥$50,000) (n=72); current smoking (n=73); depressed mood, steps/day, participant-reported walkability (n=77).

^b^ Married/common-law, annual household income (≥$50,000), current smoking (n=106); steps/day (n=118), GIS-derived walkability (n=122).
