# Supplementary material for: Neighbourhood Walkability and Daily Steps in Adults with Type 2 Diabetes
Source: PLoS One. 2016 Mar 18;11(3):e0151544. doi: 10.1371/journal.pone.0151544 (PMC4798718; doi:10.1371/journal.pone.0151544)
Supplement: S2 Table — (DOCX) [file pone.0151544.s004.docx]

**S2 Table. Characteristics of the participants that were included and excluded from the final models.**

|  | **Included^a^ (n=131)** | **Excluded^b^ (n=70)** |
| --- | --- | --- |
|  | **mean (SD)** | **mean (SD)** |
| Age, *years* | 60.5 (10.4) | 60.8 (10.9) |
| Steps/day | 5388 (2488) | 5317 (2982) |
| Body mass index, *kg/m^2^* | 30.3 (5.8) | 30.7 (5.1) |
| Diabetes duration, *years* | 9.8 (8.4) | 8.6 (7.2) |
| Years living at current address | 18.4 (12.4) | 17.5 (10.7) |
| Residential self-selection score based on active lifestyle preferences | 0 (1.0) | 0 (1.0) |
| Participant-reported walkability | 0.01 (1.5) | -0.03 (2.2) |
| GIS-derived walkability | 0.03 (2.3) | -0.1 (2.4) |
| Audit-assessed walkability | -0.001 (1.8) | 0.001 (1.6) |
| Walk Score ^®^ | 68.9 (19.2) | 63.7 (22.7) |
|  |  |  |
|  | **n (%)** | **n (%)** |
| Women | 63 (48.1) | 31 (44.3) |
| Married/common-law | 91 (69.5) | 33 (67.4) |
| University education | 50 (38.2) | 28 (40.0) |
| Annual household income, *≥ $50,000* | 53 (45.3) | 24 (39.3) |
| Ethnicity, white | 93 (71.0) | 46 (65.7) |
| Immigrant | 59 (45.0) | 34 (48.6) |
| Current smoking | 12 (9.2) | 5 (10.2) |
| Insulin use | 45 (34.4) | 21 (30.0) |
| Depressed mood | 37 (28.2) | 18 (26.1) |
| Dog ownership | 19 (14.5) | 12 (17.1) |
| Car ownership/regular vehicle access | 104 (79.1) | 58 (82.6) |
| Self-reported past participation in regular exercise | 106 (80.6) | 61 (87.0) |

^a^ Annual household income (≥$50,000) (n=117); current smoking (n=130); years living at current address (n=57); residential self-selection score based on active lifestyle preferences (n=56); steps/day (n=130).

^b^ Married/common-law and current smoking (n=49); annual household income (≥$50,000) (n=61); years living at current address and residential self-selection score based on active lifestyle preferences (n=23); steps/day (n=65); depressed mood, participant-reported walkability, GIS-derived walkability (n=69).
