# Supplementary material for: Neighbourhood Walkability and Daily Steps in Adults with Type 2 Diabetes
Source: PLoS One. 2016 Mar 18;11(3):e0151544. doi: 10.1371/journal.pone.0151544 (PMC4798718; doi:10.1371/journal.pone.0151544)
Supplement: S3 Table — (DOCX) [file pone.0151544.s005.docx]

**S3 Table. Univariate longitudinal hierarchical linear regression estimates between the covariates of interest and daily steps (n=131).^a,b^**

|  | **Change in Daily Steps (95% Credible Interval)** |
| --- | --- |
| Age, *years* | -96 (-132, -58) |
| Body mass index, *kg/m^2^* | -106 (-176, -36) |
| Diabetes duration, *years* | -36 (-85, 14) |
| Years living at current address*, years* | -5 (-54, 45) |
| Residential self-selection score based on active lifestyle preferences | 106 (-552, 761) |
| Women | -143 (-988, 673) |
| Married/common-law | 838 (-32, 1698) |
| University education | 475 (-384, 1333) |
| Annual household income*, ≥$50,000* | 382 (-480, 1233) |
| Ethnicity, *white* | -629 (-1549, 273) |
| Immigrant | 682 (-119, 1506) |
| Current smoking | 84 (-1358, 1526) |
| Regular vehicle access | -1426 (-2752, -118) |
| Insulin use | -506 (-1376, 375) |
| Absence of depressed mood | 622 (105, 1151) |
| Dog ownership | 1149 (29, 2299) |
| Spring/summer (*versus* fall/winter) | 649 (179, 1124) |
| Self-reported past participation in regular exercise | 1451 (95, 2817) |

^a^ Annual household income (≥$50,000) (n=117); current smoking (n=130); regular vehicle access (n=67); years living at current address (n=57); residential self-selection (n=56); past participation in regular exercise (n=67).

^b^ The univariate estimates for the walkability measures are reported in Table 3 of the manuscript.
