# Supplementary material for: Neighbourhood Walkability and Daily Steps in Adults with Type 2 Diabetes
Source: PLoS One. 2016 Mar 18;11(3):e0151544. doi: 10.1371/journal.pone.0151544 (PMC4798718; doi:10.1371/journal.pone.0151544)
Supplement: S4 Table — (DOCX) [file pone.0151544.s006.docx]

**S4 Table. Fully adjusted hierarchical longitudinal linear regression model for the association between participant-reported neighborhood walkability and daily steps (n=131).**

|  | **Change in Daily Steps (95% credible interval)** |
| --- | --- |
| Age, *years* | -106 (-127, -85) |
| Women | -84 (-492, 331) |
| Body mass index, *kg/m^2^* | -119 (-155, -82) |
| Absence of depressed mood | 553 (90, 1023) |
| Dog ownership | 646 (28, 1250) |
| Immigrant | 245 (-167, 655) |
| Insulin use | -305 (-762, 135) |
| Spring/summer (*versus* fall/winter) | 692 (283, 1106) |
| GIS-derived neighborhood walkability | 27 (-67, 117) |
| Participant-reported walkability |  |
| *Quartile 1* | *Reference* |
| *Quartile 2* | 103 (-457, 677) |
| *Quartile 3* | -197 (-774, 395) |
| *Quartile 4* | 1345 (718, 1976) |
